# Joint consultations in a structured GP-patient-geriatric-psychiatrist model for late-life depression: a cluster RCT

**DOI:** 10.1186/s12875-025-03002-w

**Published:** 2025-09-29

**Authors:** Lars Christian Kvalbein-Olsen, Eivind Aakhus, Ole Rikard Haavet, Ibrahimu Mdala, Erik L. Werner

**Affiliations:** 1 Department of General Practice, Faculty of Medicine, University of Oslo, Oslo, Norway; 2 The Norwegian National Centre for Ageing and Health, Vestfold Hospital Trust, Tønsberg, Norway

**Keywords:** Depression, Elderly, Late-life, General practice, GPs, Joint consultations, Collaborative care

## Abstract

**Background:**

Depression in older adults is mainly treated in general practice but is often constrained by limited resources in primary healthcare services and suboptimal access to assistance from specialized care. This study aimed to evaluate the effectiveness of a structured collaborative model between GPs and geriatric psychiatrists compared to standard follow-up for individuals aged ≥ 65 with depression.

**Methods:**

Patients with moderate depressive symptoms (Patient Health Questionnaire-9 [PHQ-9] scores of 10–19) were invited to participate in a cluster-randomized controlled trial evaluating a structured collaborative intervention model involving GPs and geriatric psychiatrists. The core component of the intervention consisted of two consecutive joint consultations with the GP, patient, and geriatric psychiatrist, supplemented by individual GP-patient consultations. PHQ-9 assessments were conducted at baseline and at 6, 12, and 18 months. The primary outcome was a ≥ 50% reduction in PHQ-9 scores.

**Results:**

35 general practitioners initially agreed to participate, yet only 19 managed to recruit one or more depressed patients. Consequently, a total of 34 patients were enrolled in the study, with 30 providing survey responses during the follow-up period for subsequent analysis. Binary analysis (≥ 50% symptom reduction) showed a greater likelihood of improvement in the intervention group compared to the control, though this difference did not reach statistical significance. Notably, both groups showed significant mean PHQ-9 score reductions (3.4 and 4.0, respectively) at 18 months, but differences in mean PHQ-9 scores between the groups across all time points were not statistically significant.

**Conclusion:**

This study did not yield significant results for the collaborative model implemented. Major challenges in the recruitment process likely contributed to the low participation rate, which may explain the absence of positive findings.

**Trial registration:**

The study was registered the 15.09.2019 in ClinicalTrials.gov with ID: NCT04078282.

**Supplementary Information:**

The online version contains supplementary material available at 10.1186/s12875-025-03002-w.

## Background

Depression in older adults is a globally widespread condition [1] with European prevalence estimated at 12% [2], and 11% within Norwegian general practice [3]. Notably, depression seems to be associated with a more substantial decline in the overall health status of patients as compared to other chronic illnesses [4]. In contrast to younger patients, depression in older patients manifests with fewer affective symptoms but more cognitive changes, diminished interest [5] anxiety [6] and somatic symptoms [5, 6]. Depression symptoms also often appear to be masked by less explicable physical symptoms, such as fatigue, diffuse pain, back pain, and chest pain, rendering depression diagnosis more challenging than in the general adult population [7].

In general practice, depression among older adults is frequently undiagnosed or untreated [3, 8]. Potential reasons for this include stigma associated with depression [9, 10] normalization of depressive symptoms as part of aging [11] and time constraints for general practitioners (GPs) who may prioritize physical health problems over mental health in consultations [12]. Furthermore, therapeutic nihilism—the belief that nothing can be done to aid this patient group—appears to be a prevalent attitude among GPs [13]. Untreated depression among older people may result in diminished quality of life, worsened chronic conditions, heightened mortality, and suicide risk [14, 15].

Depression in older adults is a treatable condition and can be managed with antidepressants, psychotherapy, and/or electroconvulsive therapy (ECT) [16, 17]. In addition, exercise and improvement of social networks are effective in alleviating depressive symptoms and may be suitable as adjunct treatment [18, 19]. Nevertheless, depression in older adults is associated with a more prolonged and chronic course, demonstrating less responsiveness to antidepressants as compared to younger patients. Somatic illness and cognitive decline in old patients appear to reduce the effectiveness of antidepressive treatment [16, 20]. 

In the current healthcare system, Norwegian GPs have the option to refer older patients to specialized care at geriatric psychiatry departments across most regions of Norway. However, the majority of GPs express a desire for increased capacity and improved collaboration with the psychiatric secondary care services [21] and when referring the patient, the GPs are often not involved until after the completion of treatment in specialist healthcare settings.

During the last two decades models of collaborative care for mental health that bridge primary health services and specialized care have shown improved outcomes compared to conventional individual treatments, also among older patients [22–26]. According to the Academy of Psychosomatic Medicine (APM) [27] a typical Collaborative Care Model comprises three core components. First, a structured tool, such as the Patient Health Questionnaire-9 (PHQ-9) for depression, is used alongside clinical consultations to identify the condition [27]. Second, a non-physician care coordinator, often a nurse, monitors symptoms, manages treatment, and coordinates care with the patient’s primary physician. Finally, a psychiatrist reviews cases with the care coordinator, providing guidance and facilitating medical treatment [27]. In Norway this have also been tried, but concerns have been raised concerning the financial challenges associated with implementing collaborative care with mental health in the Norwegian primary healthcare service [28].

An underutilized tool among GPs in Norway [28] is what is referred to as ‘joint consultation with a psychiatrist [21, 29] and experiences with this approach may lead to improved quality of care, reduced waiting times, and a potential paradigm shift for patients with mental health issues [30, 31]. Moreover, a closer collaboration between GPs and psychiatrists appears to be a sought-after intervention within the general practice community [21, 32–34].

Some countries have a psychiatry subspecialty called “Consultation-Liaison Psychiatry” (CLP) [35]with various approaches. Recent Swiss studies focused on the “joint consultation” method, where GPs in training request psychiatric evaluations for challenging cases. This involves a liaison psychiatrist assessing the patient, with the GP observing or participating, followed by a discussion and report. Feedback has been positive, as it helps GPs integrate physical and mental health concerns, enhancing patient understanding and the therapeutic relationship, as well as improving the doctor-patient relationship [31, 33]. A large Cochrane review indicated CLP is effective, especially for depression, though less so than collaborative care [34]. In Norway, a similar model placing psychologists and psychiatrists at medical centres has shown positive outcomes but is financially unsustainable due to the income system [28].

Limited evidence exists on such joint consultations for old-age depressed patients between GPs and psychiatrists both in Norway and internationally. Our objective was to develop a collaborative model within the Norwegian healthcare system that optimizes resource utilization for both GPs and geriatric psychiatrists, while leveraging their respective expertise. The concept of designating the GP as the coordinator, thereby bypassing the non-physician care coordinator, emerged as a more pragmatic and resource-efficient alternative for implementation in Norwegian contexts.

### Aims

To investigate whether patients aged 65 and older with *a probable* depression, who are offered a structured collaborative model between GPs and geriatric psychiatrists experience faster or more substantial recovery compared to standard follow-up from the GP.

## Materials and methods

### Design and setting

**Fig. 1:**
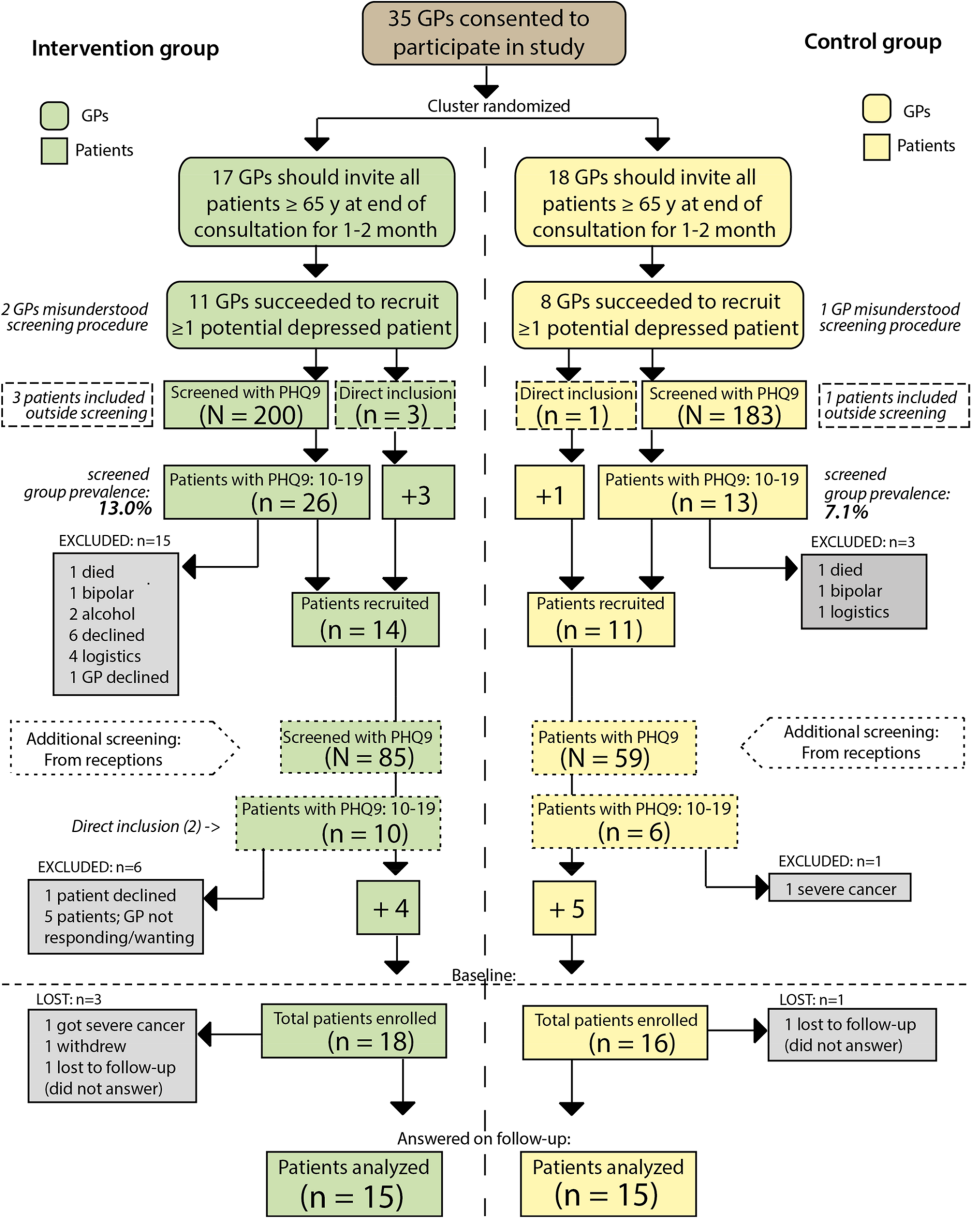
Recruitment process

Between November 2019 and June 2021, 35 GPs in southern Norway were recruited for a cluster randomized intervention study focusing on depressed older patients in general practice. Through randomization at the primary care center level, half of the GPs were assigned to implement a structured collaboration model with a geriatric psychiatrist, while the remaining GPs served as the control group. See Fig. 1 for details. The study adheres to the CONSORT guidelines (Supplementary data).

### Patient recruitment and inclusion

All patients aged 65 years and above visiting the GP in a certain period (about 2 months) were asked by the GP at the end of their consultation to respond to a specific developed questionnaire, (supplementary data 1) including on depressive symptoms, Patient Health Questionnaire (PHQ-9) [36]. This was done as a part of a linked cross-sectional study performed at the same medical centers [3]. The patients with a score ≥ 10 were invited *to* an inclusion consultation, during which the GP was required to assess the MADRS score, MMSE, and conduct a clinical evaluation to substantiate a diagnosis of clinical depression.

As this was a primary care study, we opted for a pragmatic and clinically realistic diagnostic approach, aligned with routine practice, based primarily on symptom scores and the GP’s clinical assessment rather than extensive psychiatric interviews, which would have significantly increased the burden on GPs.

Exclusion criteria included PHQ-9 ≥ 20, cognitive impairment (MMSE ≤ 21), non-native Norwegian language speakers, unhealthy alcohol use (> 14 units/week) or substance addiction, ongoing psychologist/psychiatrist treatment, significant chronic psychiatric disorders (e.g., schizophrenia, dementia, bipolar disorder), severe cancer with a limited life expectancy, and lack of patient consent. We chose to exclude individuals with a PHQ-9 score ≥ 20, as these patients typically require treatment for severe depression and standard referral.

Since recruitment for the intervention study was planned based on a preceding cross-sectional study, the lower completion rate of screening in the latter also led to a reduced identification of patients with sufficiently high PHQ-9 scores. Three GPs misunderstood the screening recruitment procedure and recruited directly patients filling inclusion criteria (Fig. 1). To address low initial recruitment, we asked staff at participating medical centres to distribute the questionnaire to all patients ≥ 65 years before their GP visit. This strategy, implemented in May–June 2021, led to a modest increase in patient enrolment (Fig. 1). Financial compensation was later offered for the added workload but had also little impact on recruitment.

### Randomization

The study was conducted as a cluster-randomized trial. The cluster comprised the general practitioner’s office and its included patients, resulting in several participating general practitioners from the same centre being grouped together. Randomization itself was performed by assigning a number to each medical centre, unknown to those conducting the randomization (one of the co-authors and an independent individual), thereby allocating approximately half of the general practitioners to the intervention group and the other half to the control group.

### Measurements and outcomes

Our main outcome was the PHQ-9 [36]. In addition to PHQ-9, patients completed Subjective Health Complaints (SHC) [37] and General Anxiety Disorder-7 (GAD-7) [38]both validated in old age [39, 40]as well as demographic data. Our primary outcome measure was treatment response, and in line with similar studies, we defined symptom reduction of 50% or more from baseline to the end of the treatment period (at 6 and 12 months) as clinically relevant response [22, 24, 41, 42]. In addition, we added an 18-month measurement to strengthen the findings.

**Fig. 2:**
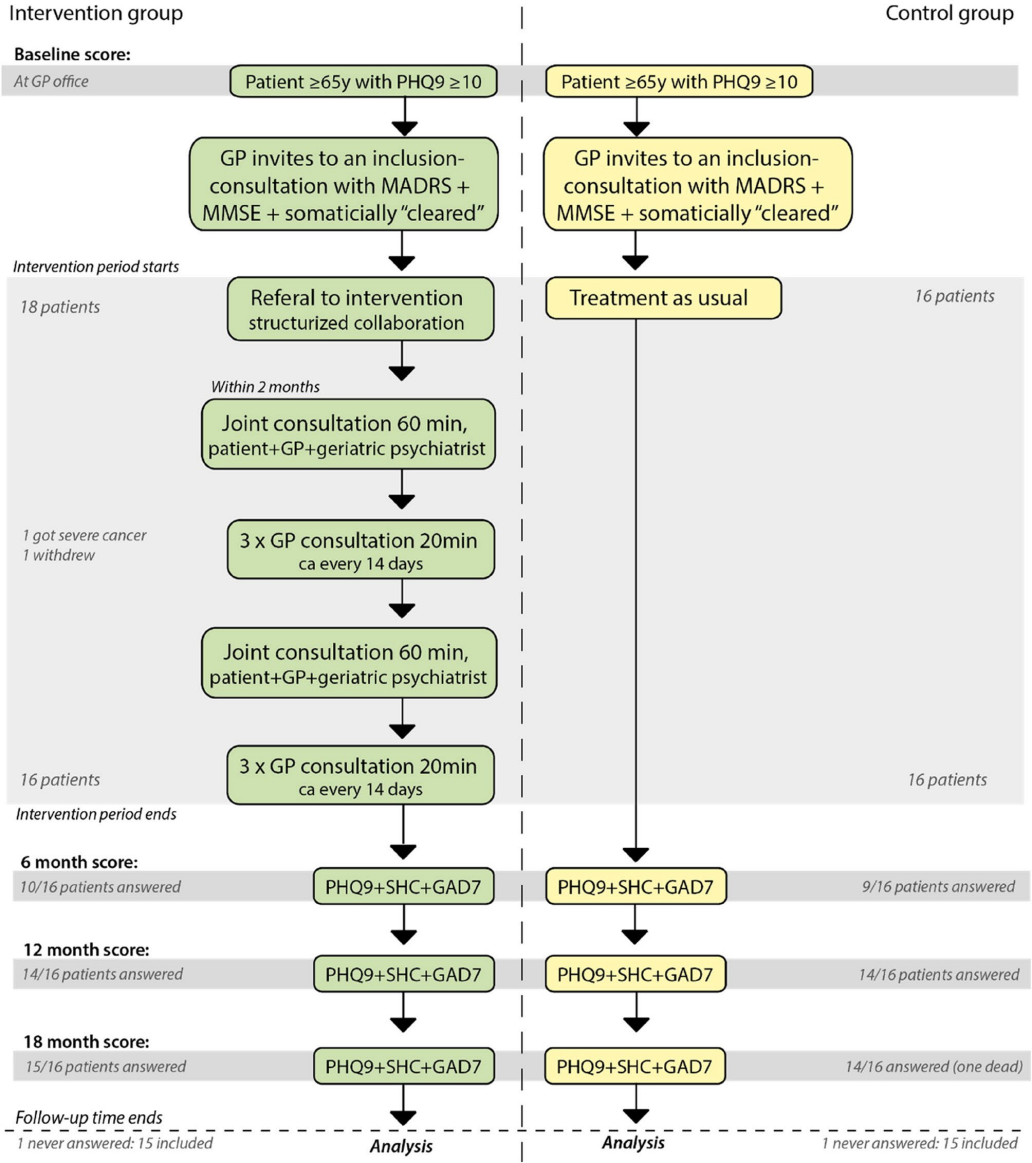
Structured collaboration model between GP and geriatric psychiatrist

Secondary outcomes were mean changes in PHQ9, SHC and GAD7 from baseline to 6, 12 and 18 months. In addition, the GPs were required to administer Mini-Mental State Examination (MMSE) [43] and Montgomery-Aasberg Depression Rating Scale (MADRS) [44] during an initial assessment for inclusion on a consultation shortly (a few weeks) after baseline. This allowed for the exclusion of individuals with a certain level of cognitive impairment, and facilitated a comparison of MADRS and baseline PHQ-9 scores [39]. Although not part of the stated objectives, we aimed to assess the performance of PHQ-9 relative to the MADRS administered by GPs (Fig. 2).

### Intervention

Upon inclusion in the intervention group, the patient was referred to two joint consultations at the local geriatric psychiatry outpatient clinic. These consultations occurred approximately three months apart, and at the first consultation with the psychiatrist, an individual treatment plan was constructed for each patient. This plan could involve medication adjustments, municipal healthcare services, social services, and other practical measures in addition to counseling with the GP. The joint consultations were followed by 2 × 3 follow-up appointments with the GP (Fig. 2).

Upon inclusion in the control group, regular depression treatment was offered by the GP. Patients in both groups received evaluation questionnaires at 6-, 12-, and 18-months post-inclusion (Fig. 2).

### Statistical methods

To calculate the sample size, we needed a reasonable assumption of improvement. As no studies have been conducted with our specific intervention, we based our estimate on the collaborative care model described in the original IMPACT study [22]. In this RCT, a 12-month response rate of 50% or more was observed in 45% of the intervention group and 19% of the control group. Using this method, with a type I error rate of α = 0.05, type II error rate of β = 0.2, and 80% power, we determined a total of 100 patients (50 × 2) were needed. Calculations were done using STATA 16.

We adjusted for clustering (design effect) and given the small number of patients per GP (estimated four), the effect was minimal. We increased the sample size by 10%, reaching 110. Adding 10% for possible lost-to-follow-ups, the final sample size should be 121, defining a need for around 60 patients per group. The 10% lost-to-follow-up estimate is low, but due to close follow-up by GPs, we expected a lower dropout rate compared to other treatment models.

We used descriptive statistics calculating frequency and percent to summarize categorical data whereas medians and range were used to summarize numerical data after checking for normality. Median differences between the study groups were assessed using the Mann-Whitney U-test whereas associations between categorical variables were established from the chi-square test.

Mixed effects linear regression models were used to assess the changes in mean PHQ score over time. Binary measures (≥ 50% PHQ-9-improvement or not) were analyzed using a binary logistic regression model. The models were adjusted for age and gender. We performed multilevel multiple imputation of the missing data using the miceadds package in R, making 10 datasets. The results of the imputed data versus complete case analyses were comparatively similar. All analyses were performed in Stata/SE 18 and the significance level was set at α = 0.05.

The study was registered in ClinicalTrials.gov with ID: NCT04078282.

## Results

Of the 35 participating GPs who screened a total of 527 patients, only 19 were able to recruit at least one patient who met the inclusion criteria. Between November 2019 and November 2021 a total of 34 patients were recruited, with 18 and 16 in the intervention and control groups, respectively. 15 patients in each group responded to one or more of the follow-up questionnaires and were included for further analysis (Fig. 2; Table 1). Despite the insufficient number of recruited participants, patient intake was concluded in November 2021 to maintain reasonable project progress. Participants who were randomized to the intervention were significantly older (median age: 77 years) compared to participants in the control group (median age: 68 years), *P* = 0.02. At baseline, there were no significant differences between the groups in median SHC, PHQ-9 and GAD (Table 1).

**Table 1:** Baseline characteristics stratified by study group

Characteristics		Study Group		
	Total included	Intervention	Control	*P*-value
Gender: n(%)				0.67
Female	24(80.0)	12(80.0)	12(80.0)	
Male	6(20.0)	3(20.0)	3(20.0)	
Total	30(100.0)	15(100.0)	15(100.0)	
Age in years: median (min, max)	74(65,88)	77(65,88)	68(65,81)	0.02
Pscychometrics: median (min, max)				
PHQ-9	13(10,19)	13(10,19)	15(10,18)	0.19
SHC	21.5(11,40)	27(13,40)	19(11,36)	0.23
GAD-7	10(1,19)	10(1,14)	10(2,19)	0.92

### Binary response (improved/not improved)

Between baseline and 6 months, 30.0% (3 of 10 patients) in the intervention group had obtained the goal of at least 50% improvement in PHQ—9 score, and none of the nine patients in the control group. Between baseline and 12 months, the rate of improvement (≥ 50%) observed in the intervention and control groups were 28.6% (4 of 14) and 14.3% (2 of 14) respectively. This gives an RR of 2.0 [95% CI: (0.43, 9.21)], for improvement in the intervention group which was not statistically significant. At 18 months from the inclusion 20% (3 of 15) had a ≥ 50% improvement from baseline score in the intervention group compared to 14.3% (2 of 14) in the control group, RR = 1.40 [95% CI: 0.27, 7.18] (Table 2).

**Table 2:** Unadjusted summary data showing the proportion of patients who showed at least 50% improvement on PHQ-9 and estimates of relative risk (RR) with 95% CI’s between study time points

		PHQ-9 ≥ 50% Improvement		
Time range	Patients, n	Intervention, n (%)	Control, n (%)	Relative risk (95% Cl)
T0 - T6	19	3/10(30.0)	0/9 (0.0)	-
T0 - T12	28	4/14(28.6)	2/14 (14.3)	2.00 (0.43, 9.21)
T0 - T18	29	3/15(20.0)	2/14 (14.3)	1.40 (0.27, 7.18)
T12- T18	27	3/14(21.4)	2/13 (15.4)	1.39 (0.28, 7.05)

### Mean changes in PHQ-9

**Fig. 3:**
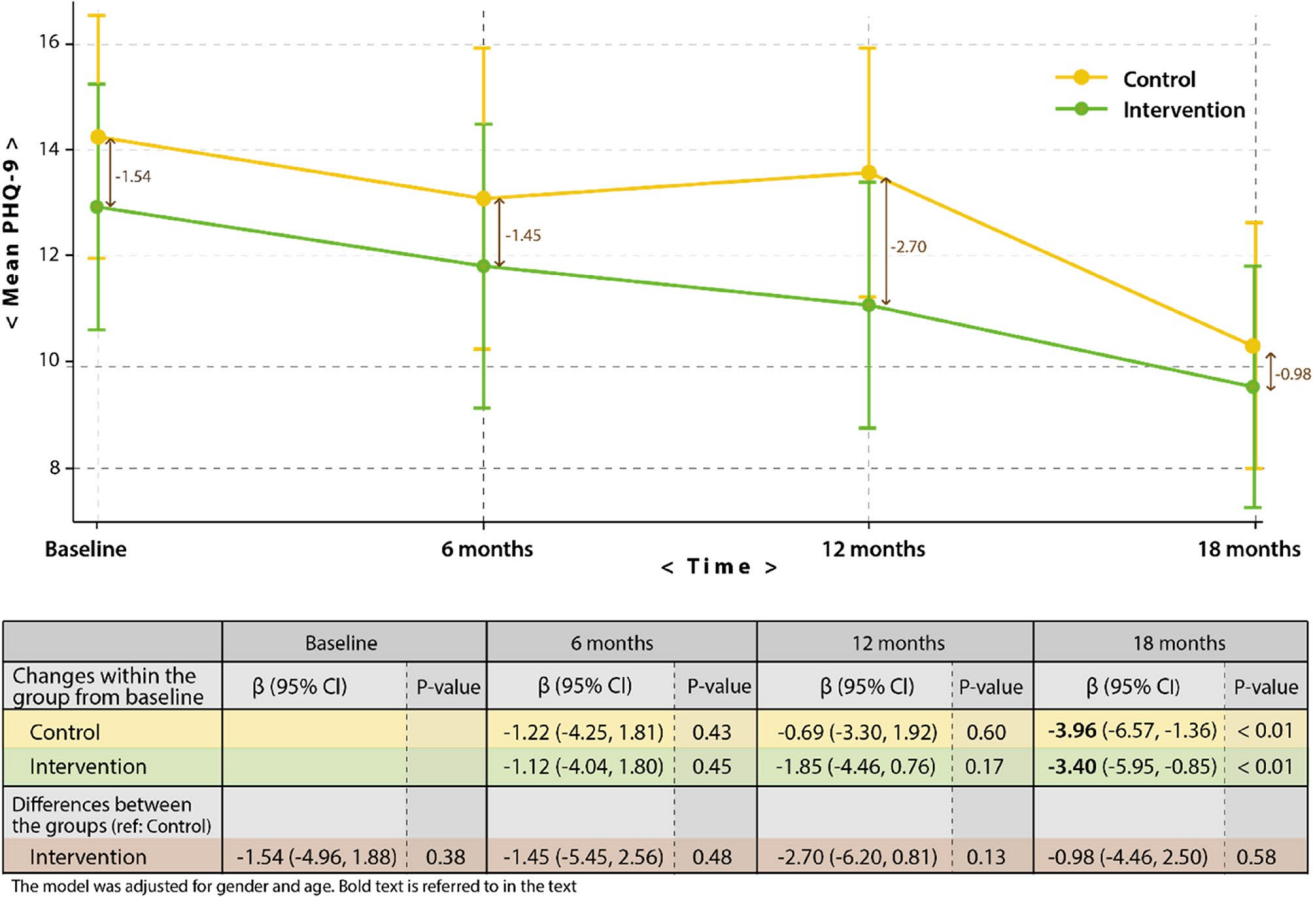
Adjusted changes in mean PHQ-9 over time within and between the groups

We found a significant decrease in PHQ-9 score of 3.40 within the intervention group and 3.96 within the control group at 18 months relative to baseline scores. Mean changes within the groups at 6 and 12 months relative to baseline values were not statistically significant. Further, mean differences in PHQ-9 scores between the groups at all study time points did not reach statistical significance (Fig. 3).

### Other findings

**Fig. 4:**
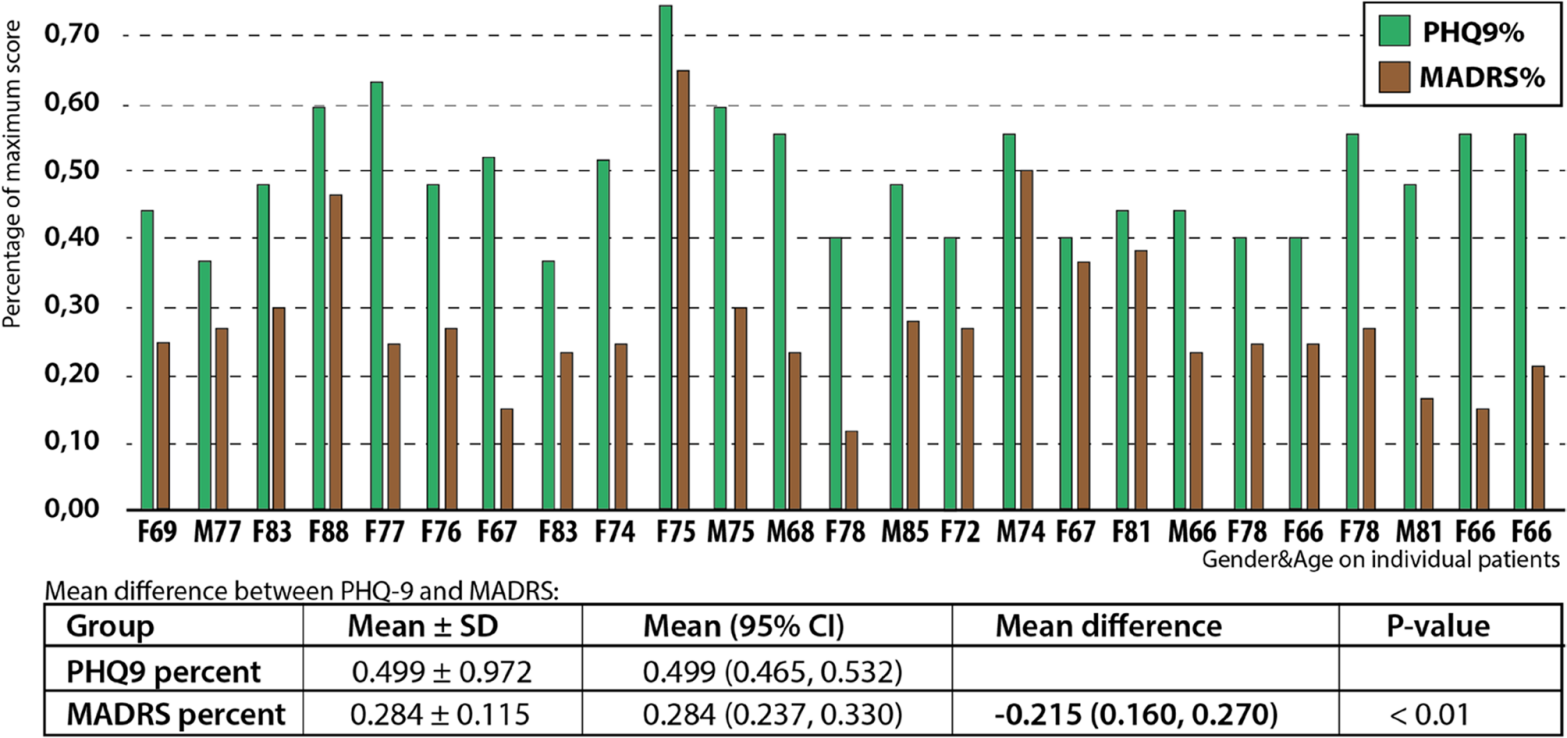
Depression assessment; Patient self-reported vs. GP-reported: PHQ-9% vs. MADRS%, and Mean difference between PHQ9% and MADRS percent

There were no statistically significant differences in mean GAD-7 and SHC scores between the groups, nor were any other variables found to differ significantly. An unexpected finding was the substantial disparity in the prevalence of moderate depressive symptoms identified during pre-recruitment screening, with the intervention group showing a prevalence of 13.0% compared to 7.1% in the control group (Fig. 1). Additionally, a notable discrepancy emerged between GPs’ MADRS scores and patients’ PHQ-9 scores. When converted to relative values of the maximum score, MADRS scores were, on average, 21.5% lower than PHQ-9 scores (*p* < 0.01, Fig. 4).

## Discussion

### Summary

Although this cluster-randomized trial did not find differences in the primary endpoint of at least 50% reduction in PHQ-9 score at 12 months following the intervention, the patients in the intervention group tended to improve faster than those in the control group. Surprisingly, outside the aims of the study, we observed a significant discrepancy between the MADRS scores assessed by the GPs and the PHQ-9 scores self-reported by the patients at inclusion, with MADRS scores generally lower than PHQ-9 scores.

### Strengths and limitations

#### Issues with recruitment

The main limitation of this study is the low recruitment rate, which also made it difficult to achieve statistically significant results. As stated in statistical methods, we originally planned this study for 120 included patients, and with only a quarter of this the possibility for showing a significant difference between the groups was very limited. Although our results show an increased likelihood of improving in the intervention group, the low number of participants made it difficult to gain any statistically significant results.

Recruitment for this study relied on a preceding cross-sectional study [3] whose limitations also affected the recruitment rate. As discussed in the analysis [3] requiring GPs to distribute a screening form at the end of consultations proved challenging, despite repeated reminders from project coordinator. Time constraints, competing medical priorities, and practical challenges often resulted in non-compliance. Surprisingly, the same issue arose when receptionists handled distribution. This highlights the need for a more robust data collection framework to ensure successful implementation without additional resources, as high workload and time pressure are simply too limiting for most GPs and medical centres.

The recruitment process was also affected by the occurrence of the Covid-19 pandemic during data collection for many of the physicians. Consequently, several patient trajectories were interrupted or not recorded or scheduled in a timely manner, particularly in 2020. This aspect also contributed to lowering the recruitment to the study.

#### Differences between the groups

Another aspect that may have influenced the outcome of the study is that patients in the intervention group were significantly older than in control. A possible reason for this may be a higher refusal rate among the younger elderly for the intervention, as it was more demanding (Fig. 1). This would not have been an issue in the control group. Age may be a negative prognostic factor regarding depression [45] and influences the presentation of depressive symptoms [46]. Furthermore, depression in older adults is associated with a more chronic course than in younger individuals [20]. Increasing age is also associated with more chronic diseases and frailty which might further reduce recovery rate in this group. On the other hand, there was no significant difference in PHQ-9 score between the groups at baseline (Table 1). However, the significantly higher prevalence of depressive symptoms in the intervention group during screening (Fig. 1) may suggest an additional population difference between the two study arms. This discrepancy is more likely due to the intervention GPs being more inclined to distribute the screening forms compared to those in the control group, leading to a potential selection bias. The direction in which this effect would influence the results remains somewhat unclear, but it could be an explanation for the higher age in the intervention group.

A greater number of participants in the intervention group either declined participation or withdrew from the study for various reasons, including logistical challenges faced by both patients and GPs, compared to the control group (Fig. 1). This may have also influenced the study results.

Perhaps those who would have had best use of the intervention declined and thereby interfered with the results in a negative way? The control group had little to gain from declining participation, aside from avoiding further assessment through questionnaires.

#### Diagnostic issues

We used a pragmatic approach to diagnostic inclusion which may have contributed to the lack of significant results. Our model included patients with moderate depressive symptoms, and these symptoms may have been caused by factors other than a primary depressive disorder. On the other hand, collaborative care has shown effectiveness in treating various mental health conditions [27]. However, it is important to note that our version of collaborative care differs significantly from the standard model and aligns more closely with a modified, repeated consultation-liaison model, which has been shown to have a greater impact on the doctor-patient relationship and a somewhat lesser effect on depression outcomes [34].

The discrepancy between the MADRS scores from the GPs and the PHQ-9 scores from the patients may suggest that GPs underestimate depressive symptoms compared to patients’ self-assessments. While this is unlikely to have influenced the lack of difference between the groups, it may have contributed to the low recruitment rate.

#### Intervention protocol issues

Additional limitations include the lack of verification regarding the GP’s adherence to the prescribed 2 × 3 follow-up GP consultations. In addition, two of the interventions were prematurely concluded following only one single joint consultation, after mutual assessments of improvement or remission by both the patient and the health care providers.

### Comparisons with existing literature

We are not alone in facing challenges with having GPs recruit patients for research on collaborative care [47]. However, as emphasized in this well-designed and significantly larger cluster-randomized controlled trial from Denmark [47] it is crucial that such unsuccessful trials are also published to provide insights and lessons for future research.

We have not identified similar intervention studies involving older patients that compare the use of joint consultations between general practitioners and geriatric psychiatrists. There have been studies on both «consultation-liaison psychiatry» [33] and on joint consultations in other psychiatric settings [31, 48] but these neither focus solely on the old-aged nor have made a direct attempt to compare with treatment as usual.

Our findings of 14.3 and 20.0% patients with recovery after 18 months in the control and intervention groups respectively align with existing research which demonstrates that 1 in 5 with old age depression experiences a functional recovery after 2 years [49].

We have previously reported from a cross-sectional study that GPs in one of three cases were unaware of their patients’ depression [3]. In line with this, we found it interesting to report the poor correlation between the GPs assessment by MADRS and the patients’ own PHQ-9 report in this study.

## Conclusions

This study failed to demonstrate that our structured collaborative model, consisting of two consecutive joint consultations involving the GP, patient, and geriatric psychiatrist, produced superior outcomes compared to standard practice in the treatment of depression in elderly patients. We believe that the low number of recruited patients and possibly differences in age between the patients in the intervention and the control group may be significant causes for this.

The evident challenges this study encountered with patient recruitment demonstrate that conducting such studies in general practice is too resource-intensive without substantial additional support. Despite the high prevalence of this condition, the optimal approach for managing and treating depression in elderly patients within the constraints of a busy GP schedule remains uncertain. Further research might investigate the factors contributing to the lack of significant differences in this study, as well as the reasons behind the low patient recruitment, despite sufficient GP participation. It is possible that older individuals may be more resistant to change or uncomfortable engaging with unfamiliar healthcare providers.

## Supplementary Information

Supplementary Material 1.

Supplementary Material 2.

## Data Availability

Due to privacy concerns the datasets is not publicly available online, but can be retrieved upon reasonable request from the corresponding author, LCKO.

## Abbreviations

GP: General practitioner
PHQ-9: Patient-Health-Questionaire-9
GAD-7: General-Anxiety-Disorder-7
SHC: Subjective-Health-Complaints
MMSE: Mini Mental Status Examination
MADRS: Montgomery-Aasberg Depression Rating Scale
CLP: Consultation-Liaison Psychiatry
