# Supplementary material for: Joint consultations in a structured GP-patient-geriatric-psychiatrist model for late-life depression: a cluster RCT
Source: BMC Prim Care. 2025 Sep 29;26:297. doi: 10.1186/s12875-025-03002-w (PMC12482570; doi:10.1186/s12875-025-03002-w)
Supplement: Supplementary file 1 — Supplementary Material 1. [file 12875_2025_3002_MOESM1_ESM.pdf]

Pas-ID: «M_01

Questionnaire for Older Patients ≥ 65 Years Attending a GP-Appointment

The purpose of this study is to learn more about the physical and mental health of older patients.
All data will be treated confidentially. Some participants may be offered further follow-up.

Please mark your answers like this: ☒

Thank you for your help.

We kindly ask you to complete all sections of the questionnaire and check both page sides.

BACKGROUND INFORMATION:

Date (today) : _______ Date of birth: ☐☐ ☐☐ / 1 9 ☐☐ Gender: Male: ☐ Female: ☐
date month year

Marital status: Married/Partnered: ☐ Widowed: ☐ Divorced: ☐ Living alone: ☐

Living situation: House: ☐ Apartment: ☐ Care facility: ☐

Did you pursue further education after primary school: Yes: ☐ No: ☐

Do you have higher education? (e.g., college, university): Yes: ☐ No: ☐

Do you have children or grandchildren nearby: Yes: ☐ No: ☐

Is Norwegian your native language? Yes: ☐ No: ☐

Do you smoke? Yes: ☐ No: ☐

Do you drink more than 2 units of alcohol per day on average? Yes: ☐ No: ☐ Don’t know: ☐
(= 3 or more half-liters of beer, glasses of wine, or shots of spirits daily)

Do you have any of the following health conditions? (Check all that apply)

Diabetes: ☐ Osteoporosis: ☐ High blood pressure: ☐ Rheumatoid arthritis: ☐

Osteoarthritis: ☐ Stroke: ☐ Cancer: ☐ Lung disease: ☐ Eye disease: ☐

Heart disease: ☐ Thyroid disease: ☐

Other chronic illness: ☐ ____ *(please specify)*

Check if you participate in or use any of the following:

Volunteer work/association/church: ☐ Paid work: ☐ Day center: ☐

Sedative medication: ☐ Sleeping medication: ☐ Antidepressants: ☐

Home nursing care: ☐ Assistance with household tasks: ☐ Psychiatric nurse: ☐

Treatment by a psychologist or psychiatrist: ☐

I agree to be contacted if further follow-up is relevant: Yes: ☐ No: ☐

FLIP PAGE, 2 SIDES

Pas-ID: «M_01

Here are some common health complaints. We ask you to evaluate each issue/symptom and indicate to what extent you have been affected by it over the past thirty days.

SHC. Ursin et al.

	(sett kun ett kryss for hver linje)	Not at all	A little	Some	Serious
1.	Cold, flu	☐ 0	☐ 1	☐ 2	☐ 3
2.	Coughing	☐ 0	☐ 1	☐ 2	☐ 3
3.	Asthma	☐ 0	☐ 1	☐ 2	☐ 3
4.	Hodepine	☐ 0	☐ 1	☐ 2	☐ 3
5.	Neck pain	☐ 0	☐ 1	☐ 2	☐ 3
6.	Upper back pain	☐ 0	☐ 1	☐ 2	☐ 3
7.	Lower back pain	☐ 0	☐ 1	☐ 2	☐ 3
8.	Arm pain	☐ 0	☐ 1	☐ 2	☐ 3
9.	Shoulder pain	☐ 0	☐ 1	☐ 2	☐ 3
10.	Migraine	☐ 0	☐ 1	☐ 2	☐ 3
11.	Extra heartbeats	☐ 0	☐ 1	☐ 2	☐ 3
12.	Chest pain	☐ 0	☐ 1	☐ 2	☐ 3
13.	Breathing difficulties	☐ 0	☐ 1	☐ 2	☐ 3
14.	Leg pain during physical activity	☐ 0	☐ 1	☐ 2	☐ 3
15.	Heart burn	☐ 0	☐ 1	☐ 2	☐ 3
16.	Stomach discomfort	☐ 0	☐ 1	☐ 2	☐ 3
17	Ulcer/non-ulcer dyspepsia	☐ 0	☐ 1	☐ 2	☐ 3
18.	Stomach pain	☐ 0	☐ 1	☐ 2	☐ 3
19.	Gas discomfort	☐ 0	☐ 1	☐ 2	☐ 3
20.	Diarrhoea	☐ 0	☐ 1	☐ 2	☐ 3
21.	Obstipation	☐ 0	☐ 1	☐ 2	☐ 3
22.	Eczema	☐ 0	☐ 1	☐ 2	☐ 3
23.	Allergies	☐ 0	☐ 1	☐ 2	☐ 3
24.	Heat flushes	☐ 0	☐ 1	☐ 2	☐ 3
25.	Sleep problems	☐ 0	☐ 1	☐ 2	☐ 3
26.	Tiredness	☐ 0	☐ 1	☐ 2	☐ 3
27.	Dizziness	☐ 0	☐ 1	☐ 2	☐ 3
28.	Anxiety	☐ 0	☐ 1	☐ 2	☐ 3
29.	Sadness/depression	☐ 0	☐ 1	☐ 2	☐ 3

Sum: _______

FLIP PAGE, 2 SIDES

Pas-ID: «M_01

Over the last 2 weeks, how often have you been bothered by the following problems?

PHQ-9

	(only one cross per line)	Not at all	Several days	More than half of the days	Nearly every day
1.	Little interest or pleasure in doing things	☐ 0	☐ 1	☐ 2	☐ 3
2.	Feeling down, depressed or hopeless	☐ 0	☐ 1	☐ 2	☐ 3
3.	Trouble falling asleep, staying asleep, or sleeping too much	☐ 0	☐ 1	☐ 2	☐ 3
4.	Feeling tired or having little energy	☐ 0	☐ 1	☐ 2	☐ 3
5.	Poor appetite or overeating	☐ 0	☐ 1	☐ 2	☐ 3
6.	Feeling bad about yourself - or that you're a failure or have let yourself or your family down	☐ 0	☐ 1	☐ 2	☐ 3
7.	Trouble concentrating on things, such as reading the newspaper or watching television	☐ 0	☐ 1	☐ 2	☐ 3
8.	Moving or speaking so slowly that other people could have noticed. Or, the opposite - being so fidgety or restless that you have been moving around a lot more than usual	☐ 0	☐ 1	☐ 2	☐ 3
9.	Thoughts that you would be better off dead or of hurting yourself in some way	☐ 0	☐ 1	☐ 2	☐ 3

Sum: _______

Over the last 2 weeks, how often have you been bothered by the following problems?

GAD-7

	(only one mark per line)	Not at all	Several days	More than half of the days	Nearly every day
1.	Feeling nervous, anxious or on edge	☐ 0	☐ 1	☐ 2	☐ 3
2.	Not being able to stop or control worrying	☐ 0	☐ 1	☐ 2	☐ 3
3.	Worrying too much about different things	☐ 0	☐ 1	☐ 2	☐ 3
4.	Trouble relaxing	☐ 0	☐ 1	☐ 2	☐ 3
5.	Being so restless that it is hard to sit still	☐ 0	☐ 1	☐ 2	☐ 3
6.	Becoming easily annoyed or irritable	☐ 0	☐ 1	☐ 2	☐ 3
7.	Feeling afraid if something awful might happen	☐ 0	☐ 1	☐ 2	☐ 3

Sum: _______

FLIP PAGE, 2 SIDES
